# Cancer-associated fibroblast activation protein in Appalachian women with uterine cervix cancer

**DOI:** 10.3389/fonc.2026.1799494

**Published:** 2026-06-01

**Authors:** Denise Fabian, Morgan S. Levy, Dava W. Piecoro, Dana Napier, Rachel W. Miller, Charles A. Kunos

**Affiliations:** 1 Department of Radiation Medicine, University of Kentucky, Lexington, KY, United States; 2 Department of Pathology and Laboratory Medicine, University of Kentucky, Lexington, KY, United States; 3 Biospecimen Procurement and Translational Pathology, University of Kentucky, Lexington, KY, United States; 4 Department of Obstetrics and Gynecology, Division of Gynecologic Oncology, University of Kentucky, Lexington, KY, United States

**Keywords:** ^212^Pb, cervical cancer, fibroblast activation protein (FAP), lead-212, radiopharmaceutical, uterine cervix cancer

## Abstract

**Introduction:**

[^212^Pb]Pb-PSV-359 is a radiopharmaceutical utilizing an alpha particle-emitting radionuclide lead-212 (^212^Pb) and a fibroblast activation protein (FAPα)-targeted antagonist being assessed as anticancer treatment. Prior nuclear imaging studies have shown that almost all cancer-associated fibroblasts within uterine cervix cancer tumors express the radiopharmaceutical target FAP when assessed by radiotracer uptake criteria. It is unknown whether immunohistopathology supports this claim. Therapeutic response to [^212^Pb]Pb-PSV-359 likely associates with FAP expression, and thus, it seems sensible to evaluate FAP immunoreactivity as a triage biomarker when intending to use this agent against persistent, recurrent or metastatic uterine cervix cancer.

**Methods:**

We examined a series of 37 uterine cervix cancer paraffin-embedded tumors to determine whether this tumor type expresses FAP at a sufficient cell proportion and staining intensity for an immunoreactive score (IRS) of six or higher, as [^212^Pb]Pb-PSV-359 is currently being evaluated in clinical trials only against tumors demonstrating radiotracer uptake criteria rather than also by immunoreactivity criteria.

**Results:**

The results show that 28 of 34 (82%) uterine cervix cancer tumors with evaluable desmoplastic stroma had cancer-associated fibroblasts expressing FAP. Twenty (59%) tumors scored at an IRS six or higher. Primary tumors from patients with stage IVB disease at diagnosis (n=11) or metastatic tumors (n=6) had cancer-associated fibroblasts expressing FAP most often (76%).

**Discussion:**

Cancer-associated fibroblast FAP immunoreactivity in this series indicates that [^212^Pb]Pb-PSV-359 radiopharmaceutical therapy might have usefulness in women with persistent, recurrent or metastatic uterine cervix cancer. A phase I clinical trial inclusive of metastatic uterine cervix cancer patients is underway (NCT06710756).

## Introduction

Women in the Commonwealth of Kentucky have the highest incidence of uterine cervix cancer in the United States (US), with women residing in its Appalachian counties contributing to this observation (11.4 per 100,000 persons [versus 7.7 per 100,000 persons in the total US], age-adjusted to the 2000 US standard population, refs (1, 2)). Women initially presenting with advanced-staged disease have an elevated risk (up to 48%) for persistent, recurrent or metastatic disease after first-line cisplatin-based radiochemotherapy (3–8). Intensifying immunochemotherapy has improved clinical outcomes despite persistent metastatic disease or treatment-stopping toxicity (9–11). A sensible next step in clinical development would assess molecularly targeted radiopharmaceuticals seeking better clinical outcomes through control of persistent, recurrent or metastatic disease.

Cancer-associated fibroblasts (CAFs), like those within uterine cervix cancer tumors, encourage replicative immortality, alter DNA damage responses through metabolic effects or soluble secreted factors and elude antitumor immunity by disturbing macrophage and T-cell lymphocyte function (12). These CAFs express fibroblast activation protein (FAP) to break down denatured types I and III fibrillar collagen, reorienting adhesion and migration of cancer cells and promoting a denser tumor microenvironment (TME) that resists immune cell penetration and anticancer drug entry (13). FAP lies almost exclusively at the cancer cell-CAF boundary such that FAP inhibitor (FAPI)-linked nuclear medicine agents can distinguish lymph nodes actually invaded by uterine cervix cancer cells from among metabolically immune-reactive lymph nodes. This is a distinction lacking in [^18^F]F-fluorodeoxyglucose positron emission tomography (14–19). While nuclear imaging studies are compelling for this disease, immunohistochemical (IHC) studies of FAP are needed to confirm these observations.

In this initial pilot study, we used FAP IHC performed on an automated IHC staining platform to report an immunoreactive score (IRS) accounting for the number of cells positive for the FAP biomarker and its intensity of stain in the stroma of uterine cervix cancer tumors. A better characterization of FAP immunoreactivity patterns in advanced-stage and metastatic uterine cervix cancers may aid in patient population triage such that clinical trials are enriched with only those patients positive for the intended molecular target of the radiopharmaceutical.

## Materials and methods

### Study material

The University of Kentucky Markey Comprehensive Cancer Center offers cancer care in a rural agricultural and urban constituency of eastern and central Kentucky (see ref (20). for demographic and county-level characterization). Uterine cervix cancer cell type (squamous cell carcinoma, adenosquamous cell carcinoma or adenocarcinoma) was assigned by standard hematoxylin-eosin examination under light microscopy. An immunoreactivity score was determined in cancer cells and in cancer-associated stroma from patients who had previously consented to future scientific study of their tumors and had their samples stored at the Biospecimen Procurement & Translational Pathology Shared Resource Facility at the University of Kentucky Markey Comprehensive Cancer Center. Thirty-two primary or metastatic site tumor samples were acquired prior to first-line therapy whereas five tumor samples of persistent (previously irradiated) disease were sampled before any second-line therapy. Thirty-seven paraffin-embedded tumors from women with FIGO staged IA2-IVB invasive uterine cervix cancer were stained for FAP and reported using an adapted IRS scoring system [**Table 1** (21)].

**Table 1:** Immunoreactivity score [IRS, adapted from ref (21)].

Percentage of cells positive (A)	Staining intensity (B)	IRS (product of A × B)
0 = no positive cells	0 = no color reaction	0-1 = negative
1 = 0-10% positive cells	1 = focal mild reaction	2-3 = mildly positive
2 = 11-50% positive cells	2 = moderate reaction	4-8 = moderately positive
3 = 51-80% positive cells	3 = intense reaction	9-12 = strongly positive
4 = 81-100% positive cells	Final IRS (A × B): 0-12	

Our primary hypothesis for this pilot study tested whether all (100%) uterine cervix cancer tumors had cancer-associated fibroblasts in stroma that had an FAP IRS score of six or higher. For this study, we made an *a priori* assumption that any FAP IRS score of sampled primary tumor of stage IVB patients equated to the FAP status of any metastasis (when the assumption truly might not be so), and these sample scores were added to the FAP IRS score results of true sampled metastases. This assumption increases our sample size but renders only preliminary descriptive analyses in this study. The Institutional Review Board at the University of Kentucky (Lexington, Kentucky, #106701) agreed to this translational oncology retrospective study.

### Immunohistochemistry

Uterine cervix cancer tumors were cut at four micrometer sections and mounted onto positively charged slides, which were baked at 58°C overnight. Immunohistochemical FAP staining was performed using the Ventana Discovery Ultra automated staining platform (Ventana Medical Systems, Tucson, Arizona, US) per the manufacturer’s instruction. Slides were deparaffinized on the instrument using EZprep Solution (Roche 950-102, Basel, Switzerland), followed by washing with Ventana Reaction Buffer (Roche 950-300), which was used for all subsequent washes. Slides then underwent on-board antigen retrieval with CC1 buffer (Roche 05424569001) using standard conditions, followed by washing and incubation with monoclonal FAP antibody (Abcam, ab207178, Waltham, Massachusetts, US) at a 1:100 dilution for 60 minutes at 37 °C. Slides were then incubated with Discovery anti-rabbit HQ (Roche 07017812001) for 20 minutes, followed by washing and incubation with Discovery Anti-HQ HRP (Roche 07017936001) for 20 minutes and visualized with Discovery Chromomap DAB (Roche 05266645001). Slides were stained off-line with Mayer’s hematoxylin and blued in ammonia water before they were dehydrated stepwise through ethanol, then cleared in two exchanges of xylene, and mounted with glass coverslips and Cytoseal XYL mounting media (Richard Allen Scientific 8312-4, Kalamazoo, Michigan, US). A positive control colon tumor tissue was included on every run to verify antibody performance.

### Microscopy and immunoreactivity score

**Figure 1:**
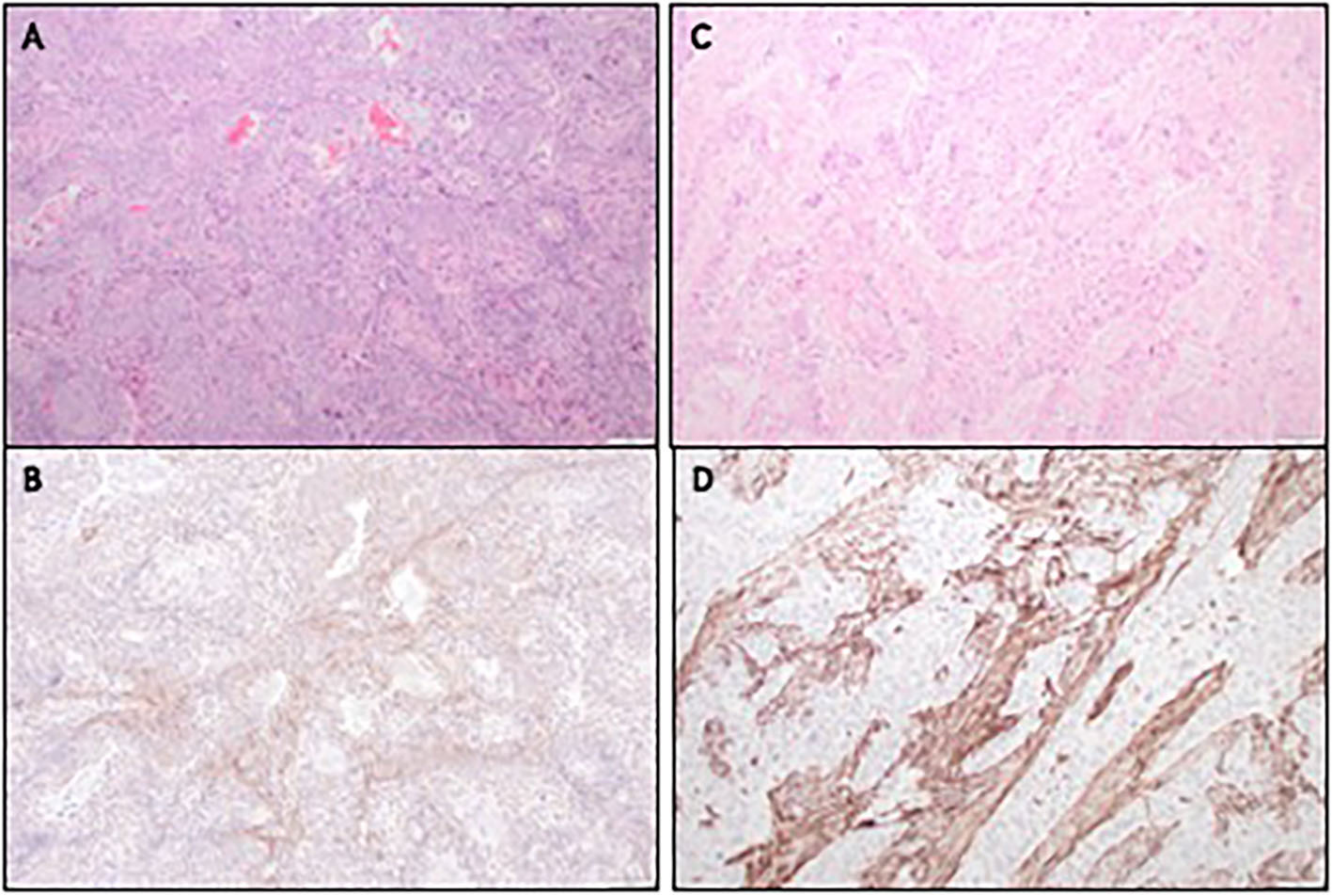
Fibroblast activation protein (FAPα) immunoreactivity in uterine cervix cancer. **(A, B)** Hematoxylin and eosin (H&E) and corresponding fibroblast activation protein (FAP) brown immunostain of uterine cervix squamous cell carcinoma showing an immunoreactivity score (IRS) of 2 (% positive score 1, stain intensity score 2). **(C, D)** H&E and corresponding FAP brown immunostain of uterine cervix squamous cell carcinoma showing an immunoreactivity score (IRS) of 12 (% positive score 4, stain intensity score 3). All images taken at 100x magnification. 10-micrometer scale bar appears in white.

Individual slides of uterine cervix cancer tumors (cells and desmoplastic stroma) were viewed on an inverted microscope (Zeiss AxioScope.A1) at 2-100x magnification (**Figure 1**). For this pilot project, one histopathologist blinded to tumor status and treatment outcome scored the percentage of cells stained and the brown staining intensity of FAP using a reporting format similar to our prior work (21, 22). As in our prior studies, the IRS is assigned in a scoring range of 0–12 because it is the multiplication product of a subjective percentage positive cells score (0–4) and a subjective staining intensity score (0–3) (**Table 1**). Digital microscopy images were acquired using the Aperio AT2 platform (Leica Biosystems) scanned at 100x magnification. Formal statistical analyses were not performed.

## Results

Thirty-seven paraffin-embedded tumors were scored—25 (68%) primary, six (16%) nodal, and six (16%) metastatic sites. Thirty-two (86%) tumors were sampled before first-line therapy; five (14%) persistent tumors were sampled prior to second-line therapy. Six (16%) patients had paired primary tumor and concurrent lymph node metastases available. Thus, a total of 31 individual patients were studied (FIGO stage at diagnosis: IA2:1, IB2:1, IIA:1, IIB:8, IIIB:2, IIIC:4, IVA:3, IVB:11). Of the 37 sampled tumors, 31 (84%) were squamous cell carcinomas (20 primary: 5 nodal: 6 metastatic) and six (16%) were adenocarcinomas (5 primary: 1 nodal).

### Uterine cervix cancer FAP immunohistochemistry

Cancer-associated fibroblasts within the stroma of uterine cervix cancer tumors most often (84%) had mild focal or more FAP immunoreactivity (IRS 2-12, **Figure 1**). No unirradiated primary or nodal uterine cervix cancer cells themselves had any FAP immunoreactivity (**Table 2**). Seventy-six percent of the patients with uterine cervix cancer metastases (stage IVB [n=11] or sampled metastases [n=6]) had FAP immunoreactivity (IRS 2-12); two women with sanctuary site metastases (ovary n=1, brain n=1) had no desmoplastic stroma and thus absent FAP immunoreactivity. FAP was immunoreactive at an IRS 6 score or higher in 20 (59%) of 34 tumors when desmoplastic stroma was present in the sample. Sixteen (47%) of these 34 samples were strongly diffusely immunoreactive (IRS 9-12). Six (18%) of 34 were assigned moderately diffusely immunoreactive (IRS 4-8). Six (18%) of 34 were classified as mildly focally immunoreactive (IRS 2-3). Six (18%) of 34 were interpreted as negative (IRS 0-1). FAP was immunoreactive at an IRS 6 score or higher in 18 (58&) of 31 squamous cell cancers; only one (20%) adenocarcinoma of the five with desmoplastic stroma available had an IRS 6 score or higher. Four (80%) of five patients with co-sampled primary and lymph node disease had concordant FAP immunoreactivity in the desmoplastic stroma of the tumor; one sample pair had no desmoplastic stroma identified in the lymph node sample and so an FAP score was not assigned. Of the five persistent uterine cervix cancer tumors after first-line radiochemotherapy, two (40%) samples had viable squamous cell uterine cervix cancer cells that themselves expressed moderate cytoplasmic and cell surface FAP immunoreactivity.

**Table 2:** Fibroblast activation protein (FAP) number and immunoreactivity score in uterine cervix cancer tumors.

Site	Cancer-associated fibroblast FAP immunoreactivity				
Uterine cervix cancer cells	Negative	Mild focal	Mild diffuse	Moderate diffuse	Strong diffuse
Primary and untreated (n = 20)	20 (100)	0 (0)	0 (0)	0 (0)	0 (0)
Nodal and untreated (n = 6)	6 (100)	0 (0)	0 (0)	0 (0)	0 (0)
Persistent and treated (n = 5)	3 (60)	0 (0)	0 (0)	2 (40)	0 (0)
Uterine cervix cancer stromal cells					
Primary and untreated (n = 19)	3 (16)	2 (11)	1 (5)	3 (16)	10 (53)
Nodal and untreated (n = 5)	1 (20)	0 (0)	1 (20)	0 (0)	3 (60)
Persistent and treated (n = 5)	1 (20)	1 (20)	1 (20)	1 (20)	1 (20)
Metastasis, untreated (n = 6)	2 (33)	1 (17)	0 (0)	1 (17)	2 (33)
Abdominopelvic (n = 3)	0 (0)	0 (0)	0 (0)	1 (33)	2 (67)
Chest (n = 1)	0 (0)	1 (100)	0 (0)	0 (0)	0 (0)
Sanctuary* (n = 2)	2 (100)	0 (0)	0 (0)	0 (0)	0 (0)

Figures in parentheses are percentages (may not total 100% due to rounding).

*Metastatic sanctuary sites are protected anatomical locations, like the ovaries (1), brain (1), eyes (0), or cerebrospinal fluid (0).

## Discussion

Our team has advocated for molecular triage when designing radiopharmaceutical clinical trials for the treatment of persistent, recurrent or metastatic uterine cervix cancer (12). We believe that patient enrichment strategies in clinical trials should limit investigational therapy to biomarker-positive patients, thereby avoiding treatment of biomarker-negative patients with therapies unlikely to be effective (23). This is an important assertion when the investigational agent is radioactive. In this translational oncology pilot study, we evaluated FAP immunoreactivity as a potential triage biomarker to identify women with uterine cervix cancer who may benefit from targeted radiopharmaceutical treatment.

Diagnostic and therapeutic FAPI radiopharmaceuticals are being tried in various cancer care settings (15–17, 24–26). Because of the proximity of CAF FAP in desmoplastic stroma to actual uterine cervix cancer cells, peptide-FAP recognition should bring DNA-damaging radionuclides directly adjacent to cancer cells, resulting in tumor cell kill. A radiopharmaceutical like [^212^Pb]Pb-PSV-359 that decays with the emission of DNA-damaging alpha particles might offer clinical benefit for this reason, and a phase I study testing the safety of that notion is underway for an assortment of cancer types (NCT06710756). When preliminary data advocate strongly that a radiopharmaceutical therapy will be ineffective in a biomarker-negative subgroup, an enrichment trial that assigns therapy only to biomarker-positive patients and assesses the effect of the therapy is appropriate. The current [^212^Pb]Pb-PSV-359 phase I trial only uses nuclear imaging criteria for an eligibility screen.

Diagnostic nuclear imaging studies of FAP report that up to 90 percent of solid tumors might be trial eligible by imaging criteria (14, 19, 25), although non-standardized radiotracer methods were used. In our study, we found that 82 percent of uterine cervix cancer tumors expressed CAF FAP. Among patients who had either metastatic disease at diagnosis (stage IVB) or those whose metastatic lesions were sampled, 76 percent of patients had uterine cervix cancer tumors expressing CAF FAP. Our findings exceed one other study where four (57%) of seven women with lymph node-positive uterine cervix cancers had IHC confirmation of FAPI-positive lesions (14). Our findings are less than that of another study of seven women with lymph node-positive uterine cervix cancers, where six (86%) lymph nodes were both IHC confirmed and [^68^Ga]Gallium-FAPI positron emission tomography and computed tomography positive (19). Further study of this association, such as in a phase 0 trial (27), remains warranted.

Whether FAP expression in uterine cervix cancer remains stable during staged tumorigenesis is unknown. By [^68^Ga]Gallium-FAPI-46 nuclear imaging criteria, up to 67 percent of premenopausal women have signal greater than physiologic background during menses (28), and so, such women that have a concurrent uterine cervix cancer might be difficult to assess for this biomarker in tumor. At the earliest stage of tumorigenesis, FAPI nuclear imaging agents have not yet been used to assess uterine cervix intraepithelial neoplasia of the uterine cervix and so no comment can be made. By [^68^Ga]Gallium-FAPI-46 positron emission tomography/computed tomography criteria, most (86%) primary uterine cervix cancers and the majority (75%) of their involved pelvic lymph nodes were detected in one series (14). While our series does not use a FAPI nuclear imaging agent, our FAP IHC fit well with this assertion, as 82 percent of primary uterine cervix cancer tumors had positive staining (IRS 2-12). Our assertion that FAP immunoreactivity in primary tumor remains the phenotype in lymph node or metastatic foci must be regarded cautiously (even though there was 80 percent concordance in our study). In future clinical trials, one translational oncology science objective might be further association of FAP IHC and FAPI nuclear imaging.

### Study limitations

Although the study was limited by a small sample size, which restricts generalizability, the qualitative approach in our study allowed for a confirmatory assessment of FAP as a radiopharmaceutical target in uterine cervix cancer fibroblasts. Our study results are conditional upon variations in pre-analytic factors such as selection/collection bias, variable pre-fixation tissue handling and archival tissue degradation from blocks stored for long periods of time. Uniformity in our biospecimen procurement, fixation and automated immunoreactivity protocols by our team lessen the impact of these pre-analytic factors upon our subsequent retrospective immunoreactivity scoring. Our study observations also are subject to high interobserver variability because of the idiosyncratic nature by which immunoreactivity scores are assigned; a centralized review by multiple experts for accuracy and consistency in FAP immunoreactivity score is not feasible here given the narrow scope of this pilot research project. Lastly, our uterine cervix cancer biospecimens were anonymized, rendering associations between clinicopathologic factors and immunoreactivity score inaccessible. An approach to overcome all these shortcomings would be to study each topic in a prospective trial with testable hypotheses and standardized biospecimen procurement/analytic protocols. Our research team has proposed a radiopharmaceutical phase II clinical trial to the United States National Cancer Institute Cancer Therapy Evaluation Program (NCI CTEP) to do just those tasks.

## Conclusion

Our study suggests that the clinical usefulness of an FAP-targeted radiopharmaceutical in the treatment of persistent, recurrent or metastatic uterine cervix cancer might be beneficial based on the high frequency of stromal FAP immunoreactivity. It remains appropriate to screen uterine cervix cancer patients for FAP immunoreactivity as an enrichment strategy for clinical trials evaluating FAPI radiopharmaceuticals.

## Data Availability

The raw data supporting the conclusions of this article will be made available by the authors, without undue reservation.
